# [Corrigendum] Goserelin promotes the apoptosis of epithelial ovarian cancer cells by upregulating forkhead box O1 through the PI3K/AKT signaling pathway

**DOI:** 10.3892/or.2025.8910

**Published:** 2025-05-07

**Authors:** Ning Zhang, Junjun Qiu, Tingting Zheng, Xiaodan Zhang, Keqin Hua, Ying Zhang

Oncol Rep 39: 1034–1042, 2018; DOI: 10.3892/or.2017.6159

Subsequently to the publication of the above paper, an interested reader drew to the authors' attention the fact that six consecutive β-actin bands looked similar, comparing across panels in Fig. 6A and B on p. 1040, even though different time points were represented in these experiments.

After having re-examined their original data files, the authors realized that the β-actin bands shown in Fig. 6A were inadvertently assembled incorrectly. The revised version of Fig. 6, now featuring the correct β-actin blots for Fig. 6A, is shown on the next page. Note that the corrections made to this figure do not affect the overall conclusions reported in the paper. The authors are grateful to the Editor of *Oncology Reports* for allowing them the opportunity to publish this further Corrigendum, and apologize to the readership for any inconvenience caused.

## Figures and Tables

**Figure 6.**
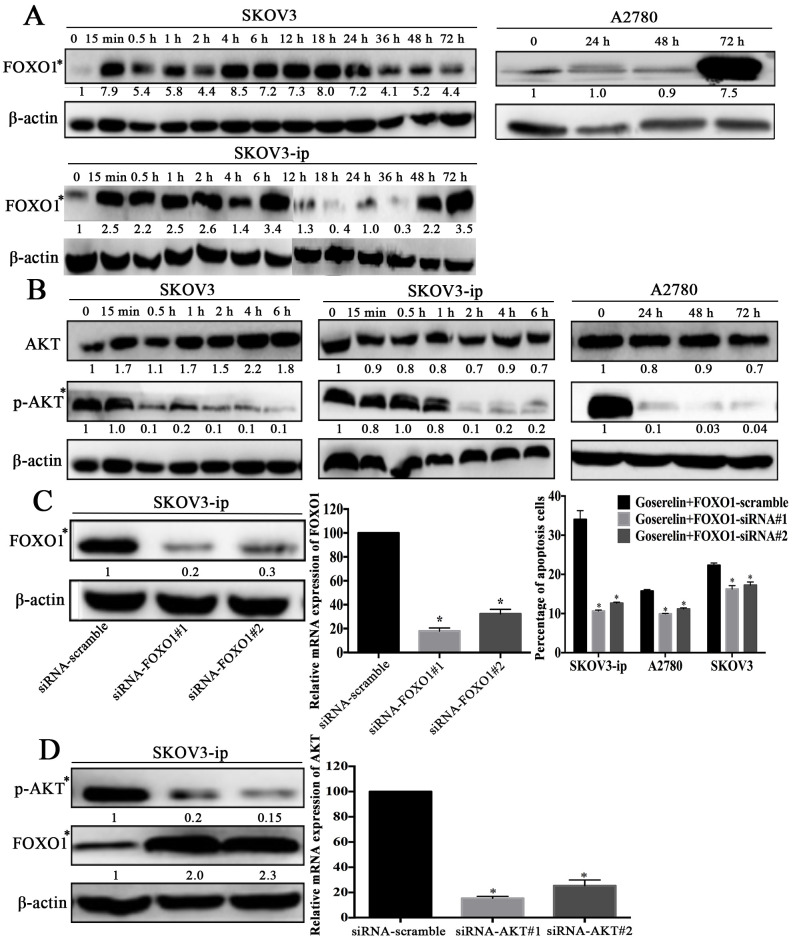
Goserelin upregulates FOXO1 through the PI3K/AKT signaling pathway. Western blot analysis of FOXO1 (A), AKT and p-AKT (B) expression in SKOV3-ip, SKOV3 and A2780 cells after treatment with 10^−4^ mol/l goserelin at different time-points. (C) Flow cytometric analysis showed that the promotion of apoptosis by goserelin was abrogated (right panel) by FOXO1-siRNA (left and middle panel). (D) Western blot analysis showed that the expression of FOXO1 was increased (left panel) by AKT-siRNA (right panel). *P<0.05, one-way ANOVA was performed for comparisons between goserelin and PBS group; t-tests were performed for comparisons between siRNA and scramble group.

